# Experimental and Numerical Research of Delamination Process in CFRP Laminates with Bending-Twisting Elastic Couplings

**DOI:** 10.3390/ma15217745

**Published:** 2022-11-03

**Authors:** Jakub Rzeczkowski, Sylwester Samborski

**Affiliations:** 1 Faculty of Technology Fundamentals, Lublin University of Technology, Nadbystrzycka 38, 20-618 Lublin, Poland; 2 Faculty of Mechanical Engineering, Lublin University of Technology, Nadbystrzycka 36, 20-618 Lublin, Poland

**Keywords:** DCB test, elastic couplings, VCCT, delamination, CFRP laminates

## Abstract

This paper aims at experimental and numerical research of delamination process in carbon/epoxy composite laminates with different fiber orientation angles in stacking sequence exhibiting the bending–twisting elastic couplings. Experimental specimens were subjected to the double cantilever beam (DCB) tests according to the ASTM D5528 regulations. Values of the mode I strain energy release rates were calculated by using three different data reduction schemes: the modified beam theory, the compliance calibration method and the modified compliance calibration. Determination of delamination initiation point was conducted in twofold way: by visual observation of crack tip using high resolution camera and by utilization of the acoustic emission technique. Numerical analyss were prepared in Abaqus/CAE Software environment by using the virtual crack closure technique (VCCT). The numerical beam model consisted of SC8R continuum shell elements. Obtained outcomes revealed that extensive fiber bridging phenomenon occurring during delamination process pronouncedly affected propagation values of strain energy release rate (*G*_Iprop_) and numerically obtained load–displacement curves. Nevertheless, in initial stage of delamination, results obtained by using the VCCT were in agreement with experimental data. The greatest value of the mode I fracture toughness equal 0.56 N/mm was obtained for the BT45 laminate.

## 1. Introduction

Delamination is a common mode of failure in composite laminates. This phenomenon can be induced easily inside composite structures or near laminate edges where three-dimensional stress state occurs. In the current literature, there is considerable amount of papers devoted to research of delamination process, both numerically and experimentally [1,2,3,4,5,6,7,8,9]. According to the fracture mechanics approach, delamination process can be analyzed by comparing fracture toughness represented by the energy release rate (ERR) at delamination interface. When unidirectional (UD) laminates are tested, a pure mode of the ERR can be determined by using procedures described by the ASTM and ISO Standards [10,11]. On the other hand, when multidirectional (MD) laminates with specific stacking sequences are involved, a mix-mode condition can be present. Hence, due to dependency of mix-modes on interface fracture toughness, decomposition of energy release rate on the mode I, II and III components is necessary [12]. Moreover, it becomes extremely important during testing laminates exhibiting elastic couplings [13,14,15] in which in-plane shear deformations and twisting curvatures cause irregularities in strain energy releases distributions along specimens width [16], as well as curved delamination front [17,18,19,20]. Elastic coupling phenomena can be described by using the classical lamination theory (CLT). In accordance with CLT, resultant moments and forces with respect to laminate’s mid-plane can be expressed by the constitutive equation given below (Equation (1)):

(1) $$\left[\begin{array}{c} N\\ M \end{array}\right]=\left[\begin{array}{cc} A & B\\ B & D \end{array}\right]\left[\begin{array}{c} \varepsilon \\ \kappa \end{array}\right]$$

where *N* is resultant force, *M* is resultant moment, A is in-plane stiffness matrix, B is coupling matrix, D is flexural stiffness matrix, *ε* is strains and *κ* is curvatures. Type of elastic coupling depends on layer configuration in composite stacking sequence. When laminate is symmetric, all components in the B matrix are equal to zero, hence the effects of elastic couplings vanish. On the other hand, when the *B*_16_ and *B*_26_ elements of coupling stiffness matrix are non-zero, then couplings between in-plane forces and twisting curvatures are induced in laminate. Moreover, non-zero components *D*_16_ and *D*_26_ introduce coupling between bending moments and twisting curvatures. These dependencies were extensively investigated by Davidson et al. [21]. They prepared finite element analysis of ERR distributions in anti-symmetric and symmetric laminates subjected to the double cantilever beam and the end-notched flexure configurations. Obtained results showed that asymmetry in ERR widthwise distributions as well as differences between minimum and maximum values of the energy release rate at crack front were the function of non-dimensional *D*_c_ and *B*_t_ parameters that were described in detail in Section 2. They also suggested that laminates should be characterized by *D*_c_ value less or equal to 0.25 to ensure uniform ERR distributions and crack front shape. In the available literature, there is a limited number of papers devoted to delamination problems in composite laminates with elastic couplings. In [22], the researchers presented numerical study of the double cantilever beam laminates exhibiting different types of elastic couplings. Results of simulations revealed that the ply stacking sequence in DCB model had a significant effect on the strain energy release rate distributions along specimens’ width. Similar results were drawn by Samborski [23]. He prepared numerical finite element analysis of the mode I SERR distributions at different boundary conditions (BCs). He concluded that initial BCs had essential influence on both the strain energy release rate widthwise distributions and the crack front shape. In [24], J. De Gracia et al. conducted analytical analysis and experimental tests of anti-symmetric and symmetric laminates. Regarding the former case, they obtained higher interlaminar fracture toughness values. It was attributed to non-uniform crack tip that was induced by the bending–twisting elastic coupling. As is stated by Sun et al. [25], curved crack front can lead to errors during experimental determination of delamination resistance. Values of the critical strain energy release rates (*G*_C_) obtained by using classical beam theory could be overestimated in this case. As underlined above, due to the fact that elastic couplings are extremely complex phenomena, their influence on delamination process should be more extensively investigated. Therefore, the current paper is devoted to experimental and numerical research of the mode I double cantilever beam laminates with elastic coupling. Novelty in the current paper is evaluation of influence of various fiber orientation angles in stacking sequence exhibiting the bending–twisting elastic couplings on the initial delamination resistance, as well as on the propagation values of the mode I strain energy release rate presented in the form of *R*-curves. Effect of fiber bridging phenomena was also investigated. Application of the acoustic emission technique in conjunction with visual recording of propagated crack tip by using high resolution camera in real time is also a new approach. This method was not used before and allows to detect delamination initiation point with high precision.

## 2. Tested Laminates

**Figure 1:**
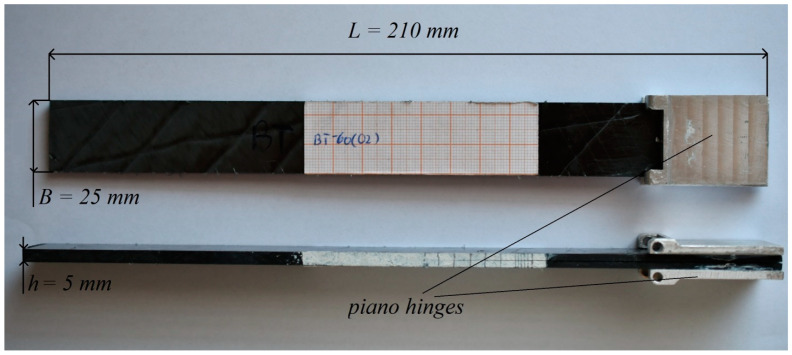
Main dimensions of experimentally tested laminates.

The DCB experimental specimens were carbon/epoxy composites with specific layup sequence consisting of 36 layers that generate the bending–twisting elastic coupling. The main dimensions of beam laminates were: total length *L* = 210 mm, width *B* = 25 mm and total thickness *h* = 5 mm, as presented in Figure 1. The pre-crack was defined by inserting a PTFE teflon foil (13 μm thick) at mid-plane between upper and lower sub-laminate. The length of film insert was 75 mm. To aid visual observation of crack propagation tip, both edges of laminates were coated with typewriter white fluid. In addition, a vertical solid line was marked in every 1 mm for the first 5 mm length and in every 5 mm for the remaining 30 mm.

Mechanical properties of CFRP laminates were obtained during tensile tests prepared in accordance with the ASTM D3039 [26] and the ASTM D3518 [27] Standards. Obtained results are collected in Table 1. In order to investigate the influence of fiber orientation angles on delamination process in coupled DCB laminates, three groups of specimens with stacking sequence [*θ* /0°/*θ*/*θ*/0°/−*θ* /0°/−*θ* /−*θ*/−*θ*/−*θ*/0°/−*θ*/*θ*/0°/0°/*θ*/*θ*] inducing the bending–twisting elastic couplings were chosen, for which the *θ* angles were {30°, 45°, 60°} (Table 2).

Effects of elastic coupling phenomena in tested laminates were assessed by calculations of non-dimensional parameters proposed by Davidson [21]. They were as follows: the bending stiffness ratio *D*_c_ and the bending–twisting coupling intensity *B*_t_. Those parameters are related to non-uniformity in strain energy release rate SERR distributions along delamination front and can be determined as a ratio of *D*_11_, *D*_12_, *D*_22_, *D*_16_ elements in the bending stiffness matrix [D]. Equations that described non-dimensional parameters are presented below:

(2) $$D_{c}=\frac{D_{12}^{2}}{D_{11}D_{22}}$$

(3) $$B_{t}=\frac{\left|D_{16}\right|}{D_{11}}$$

## 3. Experimental Procedures

**Figure 2:**
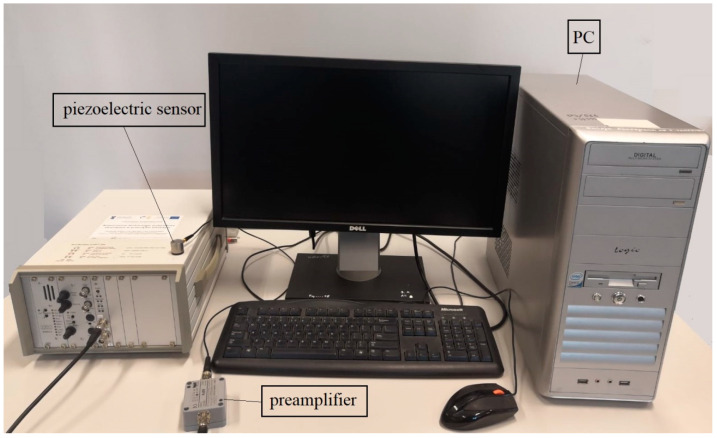
Acoustic emission test set up.

**Figure 3:**
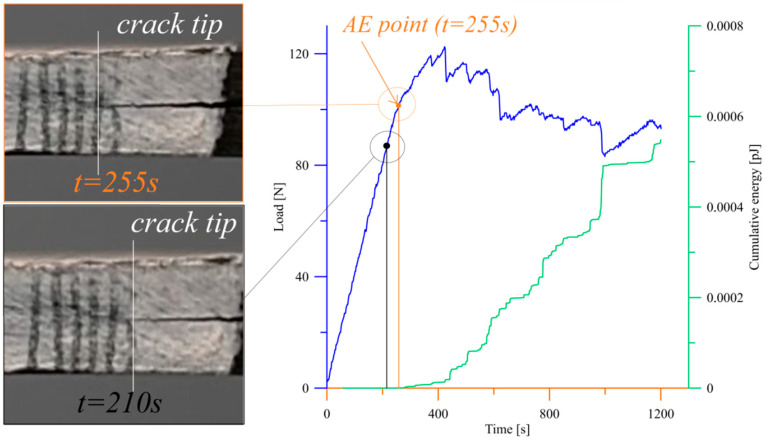
Application of acoustic emission technique combined with visual monitoring of crack tip to detection of delamination initiation point during one of the DCB test conducted on BT45 laminate.

Experimental tests were prepared on straight-sided DCB geometry specimens according to the ASTM D5528 regulations. Prior to experiments, piano hinges were bonded to laminates by using Loctite HY4070 epoxy glue. Subsequently, specimens were mounted in properly calibrated Shimadzu ASG-X tensile testing machine with sufficiently sensitive 1 kN load cell. All tests were conducted with constant crosshead velocity equal to 1 mm/min. The load–displacement responses were registered in real-time by plotting the *P*-*δ* charts. In order to monitor of crack tip and its extension during mode I fracture loading a Nikon D500 4K photo camera (Nikon, Tokyo, Japan) with set of objectives (Nikon Nikkor AF-S DX Micro 40 mm f/2.8G) and specialized led lamps for crack propagation monitoring was positioned on one side of the laminate. At first, crack tip was observed during pre-crack loading up to visually observed movement of cleavage from its initial position. Hence, the *a*_0_—initial delamination length value was determined. Afterward, a laminate was unloaded. During reloading procedure, front of growing crack was recorded in each 1 mm for the first 5 mm crack growth, and then, for every 5 mm up to 30 mm crack extension. In order to increase precision of defining the exact delamination initiation point, the acoustic emission (AE) technique was used [28]. The acoustic emission set Vallen AMSY-5 (Figure 2) supplemented with piezoelectric sensor mounted to tested laminate measured in real time various parameters of acoustic waves. The first growth of cumulative energy was treated as delamination onset and was defined as the AE point. Exemplary application of the acoustic emission technique combined with visual monitoring of crack to detection of delamination initiation point was depicted in Figure 3. In all cases, the AE points determined from cumulative energy plots corresponded to visually observed initial movement of propagated cleavage.

## 4. Analytical Determination of Energy Release Rates

In order to determine the mode I critical strain energy release rates several methods of calculation were used. Namely: the modified beam theory (MBT), the compliance calibration method (CCM) and the modified compliance calibration (MCC). The classical beam theory assumed perfectly built-in DCB beam rigidly fixed at delamination front. According to this theory the mode I fracture toughness can be expressed by following equation, where *P* is load, *δ* is load–displacement point, *B* is specimens width and *a* is current crack length.

(4) $$G_{I}=\frac{3P\delta }{2Ba}$$

In reality, the branches of double cantilever beam laminate were not perfectly clamped due to presence of initial crack region. This could cause overestimation of the *G*_I_ values. To establish unwanted deviation from classical beam theory, the correction parameter Δ, obtained experimentally, should be taken into account in data reduction scheme. Hence, the MBT method could be expressed as:

(5) $$G_{I}=\frac{3P\delta }{2B(a+\Delta )}$$

In case of the compliance calibration method, the *n* correction parameter should be determined as an exponent from the slope of log (*δ*_i_/*P*_i_) versus crack length *a*_i_. Then the CCM method is described by the equation:

(6) $$G_{I}=\frac{nP\delta }{2Ba}$$

The third method of calculation of the mode I interlaminar fracture toughness take into account a slope of the plot creating from function of cube root of compliance versus ratio *a*/*h* in the form of the *A*_1_ parameter. Therefore, values of the strain energy release rate could be determined from the following formula:

## 5. Numerical Analysis

**Figure 4:**
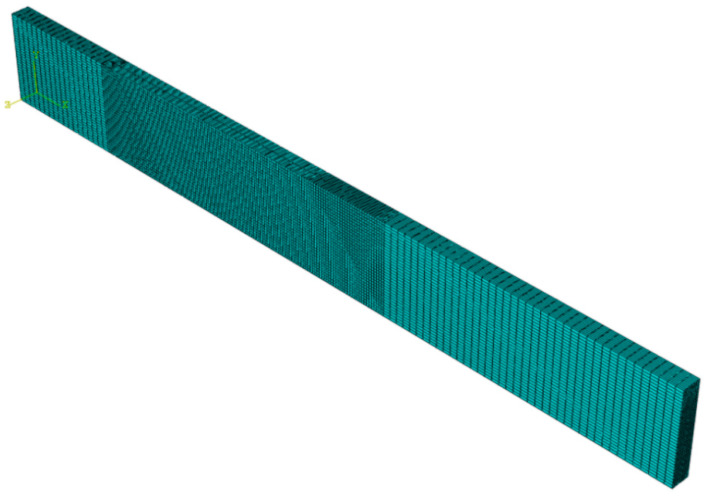
Numerical model of the DCB beam.

Numerical validations of experimental tests were prepared in the Abaqus/CAE Standard commercial finite element software. All analysis were conducted on Dell T3500 (Intel Xeon W3540 8-core processor, 2.93 GHz, 12 GB RAM, RAID-5 SATA disk matrix) workstation. During numerical simulations load versus displacement plots were obtained for bending–twisting laminates with different fiber orientation angles in particular layers. Additionally, contributions of different modes at crack front were assessed by determining strain energy release rates (SERR) widthwise distributions along specimen width. The finite element model had a form of double cantilever beam that consisted of two sub-laminates bonded together. The main dimensions of numerical DCB beam were: 210 mm total length, 25 mm width and 5 mm total thickness. In addition, whole model was divided into two branches at one beam edge on the length *a*_0_ = 45 mm which served as delamination initiator. The material properties obtained during experimental tensile tests were assigned to each layer separately. Specific stacking sequences were modeled in Abaqus/CAE composite layup module and were oriented in accordance with global coordinate system. Due to simulations were prepared on laminates exhibiting the bending–twisting elastic couplings, for which shearing deformations and twisting curvatures were expected, the FE model was built in 61050 linear hexahedral SC8R continuum shell elements in total. Unlike to conventional shell, the continuum element allow finite membrane deformations and large rotations. Moreover, the SC8R include transverse shear effects, thickness change, as well as allow for a richer transverse shear stress and force prediction. In addition, the continuum shell elements are more accurate in contact modeling than conventional shell, which is considered a big advantage. The meshed bending–twisting beam model was presented in Figure 4.

**Figure 5:**
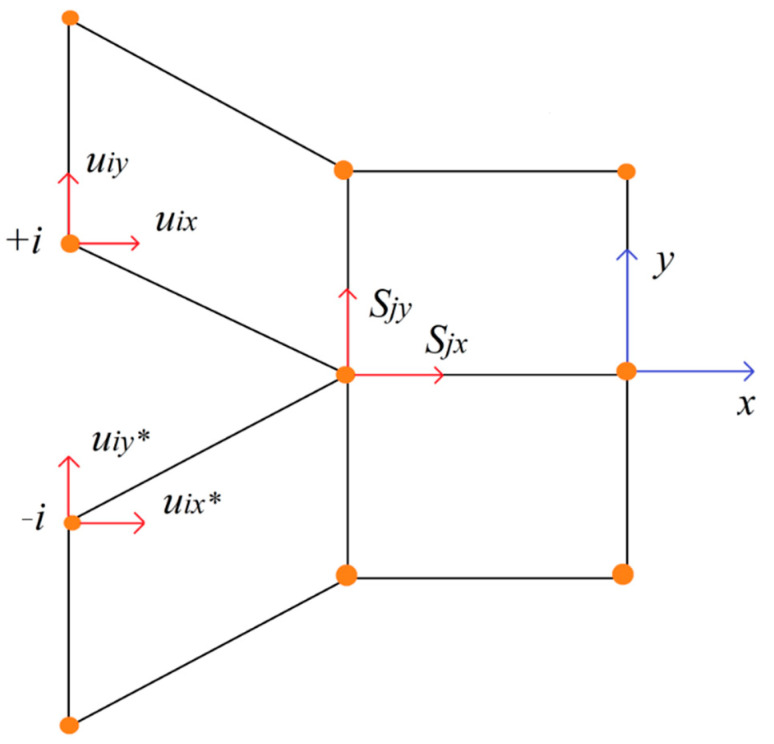
Two-dimensional representation of the VCCT technique.

In order to improve accuracy of numerical results, number of elements was increased along crack propagation path. To modeled a delamination process different methods of simulations is available such as: the cohesive zone model (CZM) [29], the continuum damage model (CDM) [30] and the virtual crack closure technique (VCCT). First method based on an exponential traction low that characterizing the behavior of the elements at the interface. The second one (CDM) utilizes stiffness degradation of adhesive elements imposed by a damage parameter. Nevertheless, for the needs of current research the origirnal virtual crack closure (VCCT) criterion, that based on linear elastic fracture mechanics, was applied. This method uses Irwin principle that assumes the change in elastic strain energy Δ*U* to be equal to the work *W*_closure_ required to close the crack. The strain energy release rate is calculated by using Clapeyron’s theory. In Figure 5 presented exemplary 2D finite element representation of the VCCT method.

The mode I (*G*_I_) and the mode II (*G*_II_) strain energy release components can be determined from Equations (7) and (8), where *S*_jx_, *S*_jy_ are forces at node *j*, *u*_iy_, *u*_ix_, *u*_iy*_, *u*_ix*_ are displacements at node *i* and Δ*a* is length of crack element.

(7) $$G_{I}=\frac{1}{2\Delta a}S_{jy}(u_{iy}-u_{iy*})$$

(8) $$G_{\mathrm{II}}=\frac{1}{2\Delta a}S_{jx}(u_{ix}-u_{ix*})$$

For pure mode I and mode II, crack growth propagation is predicted from single mode criterion. Nevertheless, as it was presented in previous research conducted by the authors [31,32,33], coupled laminates were dominated by contribution of different modes. Therefore, pure mode criterion should be replaced by mix-modes criterion that takes into account interactions between different modes. Hence, the Reeder-law (Equation (9)) was used in numerical anaylsis.

(9) $$G_{\mathrm{IC}}+\left(G_{\mathrm{IIC}}-G_{\mathrm{IC}}+G_{\mathrm{IIIC}}-G_{\mathrm{IIC}}\left(\frac{G_{\mathrm{III}}}{G_{\mathrm{II}}+G_{\mathrm{III}}}\right)\right){\left(\frac{G_{\mathrm{II}}+G_{\mathrm{III}}}{G_{T}}\right)}^{\eta }\geq 1$$

Here, the *G*_T_ = *G*_I_ + *G*_II_ + G_III_ is the total energy release rate and *G*_IC_, *G*_IIC_, *G*_IIIC_ are critical strain energy release rates in opening mode I, in-plane shear mode II and transverse shear mode III respectively. Detailed material and fracture parameters implemented to numerical analysis were collected in Table 3.

**Figure 6:**
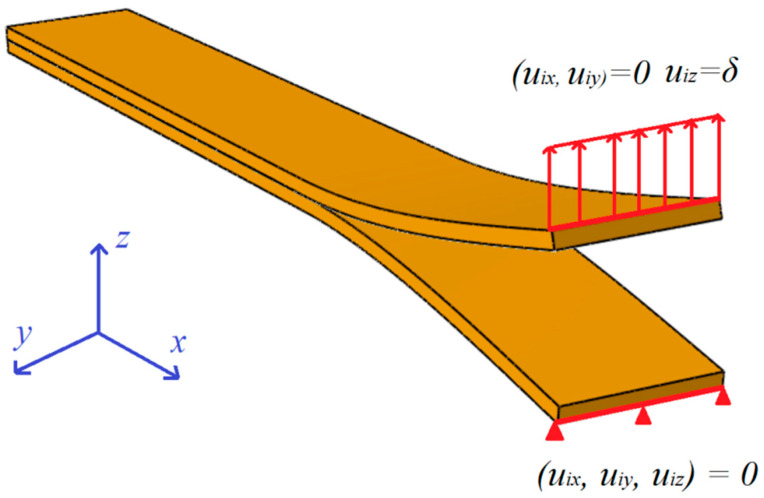
Initial boundary conditions.

Initial boundary conditions for the mode I DCB numerical model were presented in Figure 6.

For this BCs, lower sub-laminate was restrained for translation in *x*, *y* and *z* direction (*u*_ix_, *u*_iy_, *u*_iz_ = 0). In order to induce crack propagation, the displacement *δ* in *z* direction was put to the upper sub-laminate (*u*_iz_ = *δ*). Additionally, the displacement degrees of freedom in *x* and *y* direction for upper branch were also restrained.

## 6. Results and Discussion

**Figure 7:**
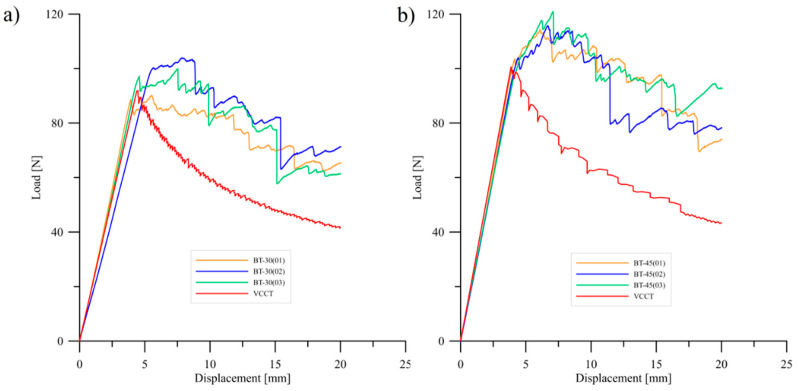
Experimental and numerical load versus displacement plots obtained for: (**a**) the BT30 and (**b**) the BT45 bending–twisting laminates.

**Figure 8:**
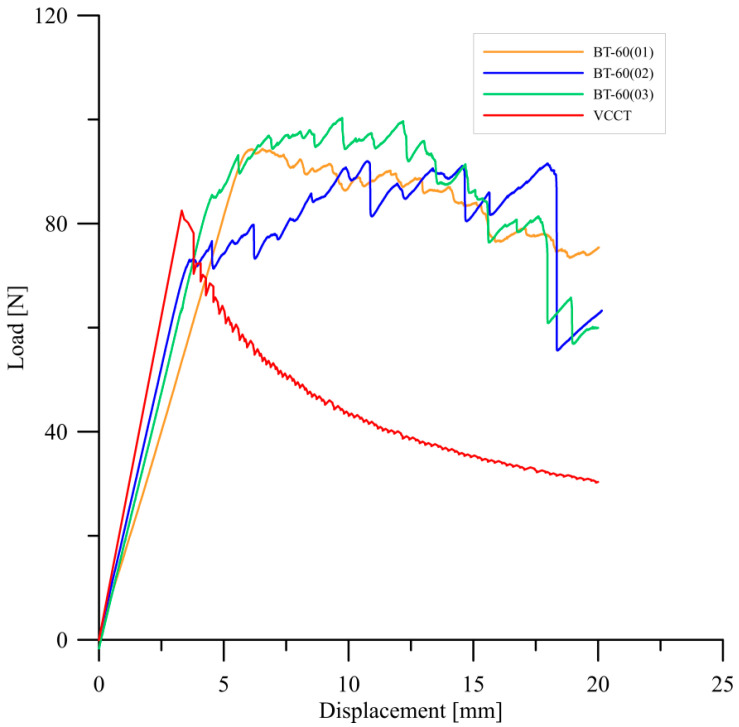
Experimental and numerical load versus displacement plots obtained for the BT60 bending–twisting laminates.

Experimental research of delamination process were conducted for three specimens in each type of bending–twisting laminates. Experiments focused on determination influence of fiber orientation angles in particular layers of BT specimens on initial values of mode I strain energy release rate values. Experimental tests were validated during finite element simulations prepared by using the virtual crack closure technique in Abaqus/CAE Software. Typical load–displacement plots obtained during DCB tests and FE analysis for different types of bending–twisting laminates are presented in Figure 7 and Figure 8. In the early stage, before delamination initiation moment, experimental and numerical curves were characterized by similar stiffness, especially for the BT30 and the BT45 laminates. In case of the BT60 specimens, these discrepancies were greater both for experimental and numerical *P*-*δ* curves. It could be caused by difficulties with precise determination of initial crack length obtained after the pre-cracking procedure. Although the BT60 specimens were characterized by the lowest values of non-dimensional parameters that describe the bending–twisting coupling intensity, differences in crack lengths on opposite sides of DCB beam ranged from 3 to 5 mm. It proved that initial pre-crack front was slightly non-uniform. Finally, it might lead to experimental and numerical errors. Considering definition of delamination initiation moment (determination of load and displacement point that was taken into consideration during experimental calculation of mode I fracture toughness), two different criteria were used. The first one was visual observation of cleavage movement from its initial position by using high-resolution camera. The second was measurement of different parameters of elastic waves generated by damage phenomena by using Vallen AMSY-5 acoustic emission system. Here, the first rapid growth of cumulative energy was taken into account as delamination onset. Owing to comparison of these two methods, it was found that delamination started to initiate when load–displacement curve started to deviate from linearity. This criterion is called the *NL* point and is clearly described in the ASTM D5528 Standard. It is also worth highlighting that because of the merging of the acoustic emission technique with monitoring of crack tip in real time, delamination onset was determined with high precision in all cases. Regarding the numerical results, it could be observed that delamination started to propagate nearly in the same point as in the experimentally load–displacement curves, in particular for bending–twisting laminates with 30° and 45° fiber orientation angles. However, for the BT60 laminate, small discrepancies between experimental and FE results were noticed. The mode I interlaminar delamination resistance values were obtained by using three different methods. Results of these calculations were collected in Table 4. The greater values of the mode I strain energy release rate determined for the BT45 laminate and were equal to approximately 0.56 N/mm. The lower values of the mode I c-SERR exhibited specimens BT30 and BT60. They were as follows: *G*_IC_ = 0.51 N/mm for the BT30 and *G*_IC_ = 0.41 N/mm for the BT60. For all types of laminates, discrepancies between calculated values of delamination resistance by using different methods were insignificant.

**Figure 9:**
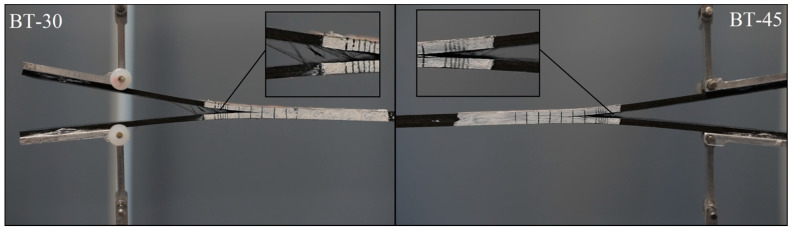
Fiber bridging phenomenon observed during DCB experiments conducted on the BT30 and the BT45 laminates.

Considering delamination propagation stage, considerable differences between experimentally and numerically obtained *P*-*δ* curves can be observed. For the latter case, with growth of applied displacement load values uniformly decreased up to *δ* = 20 mm. For the experimentally tested DCB specimens, load trends were affected by the effect of fiber bridging. In all cases, the differences between experimental and FE plots were around 30 N. In addition, sharp load jumps were observed. This phenomenon was related to merging of singular fibers to the bundles. During debonding of such bundles, a large quantity of energy released that produced observable violent loads drop. Effect of fiber bridging was observed during experimental research for all tested laminates. One of the examples of this phenomenon is presented in Figure 9.

**Figure 10:**
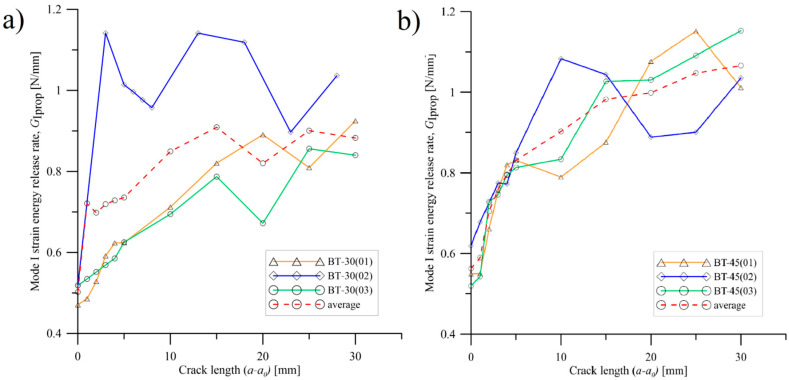
*R*-curves obtained for (**a**) the BT30 and (**b**) the BT45 laminates.

**Figure 11:**
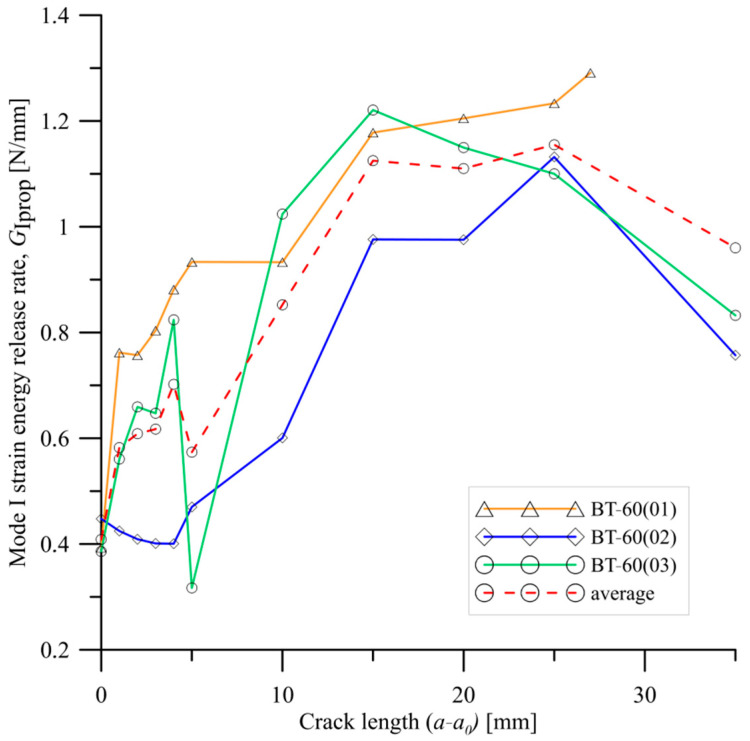
*R*-curves obtained for the BT60 laminates.

As it was proved in [34], extensive fiber bridging strongly affected the values of load and fracture energy during crack propagation. In this work, de Moura et al. proved that bridging phenomenon was responsible for up to 60% of energy being dissipated during delamination process. It was assessed by mechanical cutting of bridged fibers when crack propagated during opening–loading. This procedure allowed them to obtain a good agreement between the experimental and numerical load–displacement curves. With reference to current research, it could explain the differences in *P*-*δ* plots obtained for elastically coupled laminates. Propagation values of fracture toughness (*R*-curves) were presented in Figure 10 and Figure 11. The mode I SERR, *G*_Iprop_, were obtained based on experimental data by using the modified beam theory. For initial propagation stage (0 mm < *a-a_0_* < 10 mm), nearly for all cases, propagation values of fracture toughness were in the range between 0.4 N/mm and 0.8 N/mm. With growth of crack length, strain energy release rates significantly increased, reaching values from 0.8 N/mm up to 1.3 N/mm. In case of the BT60 laminates, for crack length between 15 mm and 25 mm, a short plateau could be observed. Here, the *G*_Iprop_ values were in the range between 0.9 N/mm and 1.1 N/mm. Preferable considerations about mode I propagation fracture toughness could be conducted by comparison average values of SERR obtained for BT30, BT45 and BT60 laminates. It could be noticed that for BT30 specimens, average values of *G*_Iprop_ started stabilizing on the level 0.85 N/mm at crack length equal to 15mm. Similar situation can be observed for BT60 laminate, but in this case, the mode I SERR values reached greater level around 1.1 N/mm. On the other hand, for BT45 specimens, propagation fracture toughness values seemed to continuously grow and any pronounced plateau was not observed. Significant differences in mode I strain energy release rate propagation values could mainly come from unstable delamination propagation caused by extensive fiber bridging.

**Figure 12:**
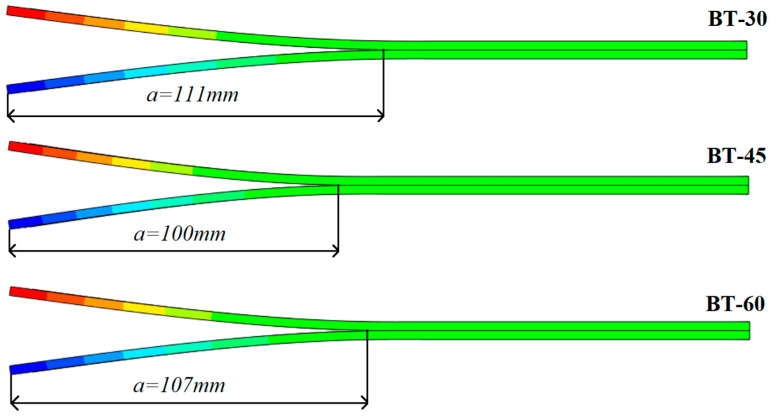
Total crack lengths obtained during the FE simulations conducted on different types of BT laminates.

Effect of this phenomenon was substantiated by comparing differences between total propagation crack lengths obtained experimentally as well as numerically. Note that for both cases applied displacement in *z* direction was 20 mm. Figure 12 presents entire crack lengths determined during finite element simulations conducted on various types of bending–twisting specimens. With regard to experimentally obtained delamination lengths, values of *a* were as follows: for the BT30 (*a* = 85 mm), for the BT45 (*a* = 75 mm) and for the BT60 (*a* = 78 mm). To sum up, in all cases, total real crack lengths were around 30 mm shorter than those obtained numerically.

The last part of numerical research covered evaluation of contributions of mix-modes in delamination process. In Table 5, the collected ratio of singular modes versus total fracture toughness is presented in percentage. It should be underlined that for all elastically coupled laminates contribution of the mode I was around 99%. The remaining 1% related to the mode II and III fracture schemes reaching the greatest values near beam model edges. Such small percentage contributions of the in-plane shear and the transverse shear modes were not expected in case of laminates with bending–twisting couplings. On the other hand, restrained displacements of upper branch in *x* and *y* directions could enforce global mode I circumstances. In reality, global mode I conditions were obtained by fixing piano hinges in tensile testing machine grips.

## 7. Conclusions

Mode I experimental and numerical research were conducted on carbon/epoxy composite laminates exhibiting the bending–twisting elastic couplings. Three groups of specimens with different fiber orientation angles were distinguished, namely BT30, BT45 and BT60. Experimental tests were prepared according to recommendations described in the ASTM D5528 Standard. Combination of visual recording of crack tip with acoustic emission technique ensured high precision of detecting the initiation delamination point. Values of critical strain energy release rate were determined by using three different methods of calculations: the direct beam theory, the compliance calibration method and the modified compliance calibration. Numerical simulations were prepared in Abaqus/CAE environment by using the virtual crack closure technique. The DCB model was built from SC8R continuum shell elements. Obtained outcomes revealed that:

- The greatest value of the *G*_IC_ equal to 0.56 N/mm was obtained for BT45 laminate. On the other hand, the lowest mode I c-SERR determined for BT60 laminate was equal to 0.41 N/mm. In case of the BT30 specimens, the mode I fracture toughness reached 0.51 N/mm. Moreover, the differences between delamination resistance values determined by using three different methods were slight.
- For all tested laminates, an extensive fiber bridging was observed during delamination process. It influenced the unstable crack propagation and the discrepancies in propagation strain energy release rate values.
- Numerically obtained load versus displacement plots were in agreement with experimental data only in initial stage of delamination. For propagation stage, the fiber bridging phenomenon influenced on differences between numerically and experimentally obtained *P*-*δ* curves. Nevertheless, application of the VCCT technique to simulate delamination in elastically coupled laminates was found out to be a sufficient tool, in particular for modeling the initial stage of delamination.
- Prevailing contribution of the mode I fracture scheme in delamination process was affected by the constraints imposed by the piano hinges.

To conclude, elastic couplings strongly influenced the mode I delamination process. Therefore, it is important to recognize the effect of these phenomena on delamination under the in-plane shear and especially under the transverse shear fracture modes. It seems to be still unexplored area of mechanics of composite materials and will be an object of future research.

## Figures and Tables

**Table 1:** Material properties of carbon/epoxy composite laminates.

*E*_1_ [GPa]	S.D. [%]	*E*_2_ [GPa]	S.D. [%]	ν_12_	S.D. [%]	*G* [GPa]	S.D. [%]
112.105	3.56	7.421	2.61	0.27	8.28	3.338	2.18

**Table 2:** Experimental elastically coupled laminates with different fiber orientation angles.

Specimens	Stacking Sequence (for One Branch)	*B*_t_ [-]	*D*_c_ [-]
BT30	30/0°/30/30/0°/−30/0°/−30/−30/−30/−30/0°/−30/30/0°/0°/30/30	0.2238	0.2208
BT45	45/0°/45/45/0°/−45/0°/−45/−45/−45/−45/0°/−45/45/0°/0°/45/45	0.2363	0.2384
BT60	60/0°/60/60/0°/−60/0°/−60/−60/−60/−60/0°/−60/60/0°/0°/60/60	0.1341	0.0990

**Table 3:** Fracture and material parameters implemented during FE simulations.

	E_11_ [MPa]	E_22_ = E_33_ [MPa]	G_12_ = G_13_ [MPa]	G_23_ [MPa]	ν_12_ = ν_13_ [-]	ν_23_ [-]	G_IC_ [N/mm]	G_IIC_ [N/mm]	G_IIIC_ [N/mm]	η [-]
BT30	112,105	7421	3338	2769	0.27	0.34	0.51	0.7	0.7	1.685
BT45							0.56			
BT60							0.41			

**Table 4:** Values of critical strain energy release rates determined for bending–twisting coupled laminates by using different data reduction schemes.

Laminate	BT30			BT45			BT60		
	*G*_IC_ [N/mm]			*G*_IC_ [N/mm]			*G*_IC_ [N/mm]		
	CCM	MBT	MCC	CCM	MBT	MCC	CCM	MBT	MCC
1	0.48	0.48	0.47	0.56	0.55	0.55	0.39	0.39	0.39
2	0.52	0.52	0.54	0.62	0.62	0.62	0.45	0.45	0.45
3	0.53	0.52	0.52	0.53	0.52	0.51	0.39	0.39	0.39
average	0.51	0.51	0.51	0.57	0.56	0.56	0.41	0.41	0.41

**Table 5:** Mix-modes contributions.

	BT30	BT45	BT60
*G*_I_/*G*_T_	99 %	99%	99%
*G*_II_/*G*_T_	0.6%	0.8%	0.85%
*G*_III_/*G*_T_	0.4%	0.2%	0.15%

## Data Availability

Not applicable.
